# Predicting Glycemic Control in Patients With Impaired Fasting Glucose With Fasting Respiratory Exchange Ratio

**DOI:** 10.1210/jendso/bvaf047

**Published:** 2025-03-15

**Authors:** Andrea Foppiani, Federica Sileo, Francesca Menichetti, Giorgia Pozzi, Silvia Gallosti, Ramona De Amicis, Alessandro Leone, Simona Bertoli, Alberto Battezzati

**Affiliations:** Department of Food, Environmental and Nutritional Sciences (DeFENS), International Center for the Assessment of Nutritional Status and the Development of Dietary Intervention Strategies (ICANS-DIS), University of Milan, Milan 20133, Italy; Department of Endocrine and Metabolic Diseases, IRCCS Auxologico, Clinical Nutrition Unit, Milan 20145, Italy; Department of Food, Environmental and Nutritional Sciences (DeFENS), International Center for the Assessment of Nutritional Status and the Development of Dietary Intervention Strategies (ICANS-DIS), University of Milan, Milan 20133, Italy; Department of Endocrine and Metabolic Diseases, IRCCS Auxologico, Clinical Nutrition Unit, Milan 20145, Italy; Department of Food, Environmental and Nutritional Sciences (DeFENS), International Center for the Assessment of Nutritional Status and the Development of Dietary Intervention Strategies (ICANS-DIS), University of Milan, Milan 20133, Italy; Department of Food, Environmental and Nutritional Sciences (DeFENS), International Center for the Assessment of Nutritional Status and the Development of Dietary Intervention Strategies (ICANS-DIS), University of Milan, Milan 20133, Italy; Department of Food, Environmental and Nutritional Sciences (DeFENS), International Center for the Assessment of Nutritional Status and the Development of Dietary Intervention Strategies (ICANS-DIS), University of Milan, Milan 20133, Italy; Department of Food, Environmental and Nutritional Sciences (DeFENS), International Center for the Assessment of Nutritional Status and the Development of Dietary Intervention Strategies (ICANS-DIS), University of Milan, Milan 20133, Italy; Department of Endocrine and Metabolic Diseases, IRCCS Auxologico, Laboratory of Nutrition and Obesity Research, Milan 20145, Italy; Department of Food, Environmental and Nutritional Sciences (DeFENS), International Center for the Assessment of Nutritional Status and the Development of Dietary Intervention Strategies (ICANS-DIS), University of Milan, Milan 20133, Italy; Department of Endocrine and Metabolic Diseases, IRCCS Auxologico, Clinical Nutrition Unit, Milan 20145, Italy; Department of Food, Environmental and Nutritional Sciences (DeFENS), International Center for the Assessment of Nutritional Status and the Development of Dietary Intervention Strategies (ICANS-DIS), University of Milan, Milan 20133, Italy; Department of Endocrine and Metabolic Diseases, IRCCS Auxologico, Laboratory of Nutrition and Obesity Research, Milan 20145, Italy; Department of Food, Environmental and Nutritional Sciences (DeFENS), International Center for the Assessment of Nutritional Status and the Development of Dietary Intervention Strategies (ICANS-DIS), University of Milan, Milan 20133, Italy; Department of Endocrine and Metabolic Diseases, IRCCS Auxologico, Clinical Nutrition Unit, Milan 20145, Italy

**Keywords:** fasting respiratory exchange ratio (RER), impaired fasting glucose (IFG), metabolic flexibility, glucose metabolism, lifestyle intervention, fat oxidation

## Abstract

**Context:**

Impaired metabolic flexibility is associated with prediabetes. However, its assessment with reference methods is impractical in routine clinical practice.

**Objective:**

This study investigates the relationship between fasting respiratory exchange ratio (RER), measured through indirect calorimetry, and glucose metabolism in individuals with prediabetes.

**Methods:**

The study involved 2 cohorts: (1) a cross-sectional cohort of 10 176 individuals to assess the association between fasting RER and glucose metabolism parameters, and (2) a matched longitudinal cohort of 86 patients with impaired fasting glucose, categorized into fat oxidation (RER < 0.775) and glucose oxidation (RER > 0.925) groups, to evaluate the impact of fasting RER on impaired fasting glucose resolution and fasting glucose after a 1-year lifestyle intervention.

**Results:**

In the cross-sectional cohort, a higher fasting RER was associated with higher fasting glucose, insulin, and Homeostatic Model Assessment for Insulin Resistance. In the longitudinal cohort, the fat oxidation group showed a greater reduction in fasting glucose (+5.9; 95% CI 1.4, 10; *P* = .011) and a higher probability of achieving normal fasting glycemia (log(odds ratio) −0.89; 95% CI −1.8, −0.03; *P* = .046) after the intervention, despite similar weight loss between groups.

**Conclusion:**

Our findings suggest that fasting RER, a readily accessible clinical measure, can provide valuable insights into glucose metabolism and impaired fasting glucose resolution. A lower fasting RER, indicative of a greater capacity for fat oxidation, is associated with improved glycemic control after a lifestyle intervention.

Metabolic flexibility is the organism's adaptive capacity to adjust its fuel oxidation in response to fluctuations in fuel availability. It is the body's ability to efficiently switch between energy sources, primarily carbohydrates and fats, based on physiological circumstances or demands [1].

Impaired metabolic flexibility may be a key contributor to the development and progression of metabolic syndrome. Indeed, metabolic inflexibility and insulin resistance are tightly related [2]. As reviewed by Goodpaster and Sparks [3], the inability to efficiently utilize fats as a fuel source leads to an increased reliance on glucose, contributing to hyperglycemia and increasing the risk of type 2 diabetes. Impaired fat oxidation, coupled with excessive caloric intake, can promote fat accumulation, particularly in the abdominal region, a key characteristic of metabolic syndrome. Consequently, individuals with reduced metabolic flexibility face an elevated risk of cardiovascular disease, type 2 diabetes, and other complications associated with metabolic syndrome.

Metabolic flexibility is not easy to assess in clinical practice. Prior studies have combined a hyperinsulinemic–euglycemic clamp with indirect calorimetry to study metabolic flexibility under resting conditions [4]. It involves infusing human insulin to assess insulin sensitivity, while simultaneously measuring substrate utilization via indirect calorimetry. This method provides a direct assessment of insulin sensitivity and the capacity to switch from fat to carbohydrate oxidation. Although more recently indirect calorimetry alone has been used to assess metabolic flexibility of subjects undergoing a VO_2_ max test, an indicator of cardiovascular fitness and aerobic endurance, on a cycloergometer [5].

Fasting indirect calorimetry is widely used in the field of nutrition, allowing for precise and personalized assessment of an individual's metabolic rate, but it can also be used to analyze substrate utilization. The respiratory exchange ratio (RER) is calculated as the ratio of carbon dioxide produced (VCO_2_) to oxygen consumed (VO_2_) during indirect calorimetry. As different fuel sources have distinct RER values, by measuring RER one can estimate the proportion of carbohydrates and fats being oxidized for energy. A higher RER (closer to 1.0) signifies greater carbohydrate utilization, while a lower RER indicates a predominance of fat oxidation [6].

An overnight fast typically results in a RER of 0.80 to 0.85, signifying a predominant reliance on fat oxidation for energy production. This shift is due to hormonal changes and substrate availability [7, 8]. Nonetheless, individual fasting RER values may vary depending on factors like prior diet, metabolic health, and physical activity [1].

This study investigates the relationship between fasting glucose metabolism and fasting RER. We aim to:

1. Explore the utility of fasting RER as an indicator of metabolic flexibility, despite its inherent limitations. We hypothesize that individuals with higher fasting RER, indicative of reduced fat oxidation, will exhibit similar glucose metabolism derangements as those observed in metabolically inflexible individuals.
2. Evaluate the prognostic value of fasting RER in predicting the resolution of impaired fasting glucose and the degree of fasting glucose improvement in response to a lifestyle intervention. We anticipate that individuals with lower fasting RER, suggesting a greater capacity for fat oxidation, will experience greater improvements in fasting glucose after the intervention.

## Materials and Methods

### Study Design, Setting, and Participants

To address aim 1, we conducted a cross-sectional cohort study. Participants were self-referred individuals seeking a weight loss program at our center. Each participant underwent a comprehensive nutritional assessment, including anthropometric measurements, indirect calorimetry, a clinical evaluation, and laboratory examinations.

For aim 2, we used a matched longitudinal cohort design: patients with impaired fasting glucose following a prescribed hypocaloric diet with an RER indicative of glucose oxidation metabolism during fasting were matched to patients with an RER indicative of fat oxidation metabolism during fasting. Secondary endpoints were body weight, body mass index (BMI), waist circumference, and body fat percentage.

Both groups received a standardized diet with the following characteristics: caloric intake matched to resting energy expenditure; protein content aligned with the Italian recommended daily allowances [9]; similar percentages of carbohydrates (50.5% on average, of which 14.8% from sugars) and fats (30.5% on average); and similar fiber content (17.5 g/1000 kcal on average).

This standardization of macronutrient composition aimed to minimize the potential influence of dietary differences on the observed outcomes.

The study was conducted at the International Center for the Assessment of Nutritional Status (ICANS, University of Milan, Milan, Italy), as part of a large ongoing open-cohort nutritional study. Patients were recruited between January 2017 and January 2019. At baseline, patients received a full nutritional assessment; based on the assessment, a hypocaloric diet was provided, and a follow-up examination was scheduled. At follow-up, patients were interviewed by a registered dietitian, and the main and secondary outcomes were measured. In this ongoing cohort study, we evaluated the 1-year follow-up examination.

Patients included in this study were self-referring patients seeking a weight loss program, mainly resident in Milan or nearby cities. For aim 2, patients were required to have impaired fasting glucose (fasting glucose >100 mg/dL and <126 mg/dL) [10]. Eligibility criteria were age ≥18 years; not pregnant nor breastfeeding; no condition severely limiting movements and physical activity; no severe cardiovascular, neurological, endocrine, or psychiatric disorder (any condition that, in the opinion of the investigators, would preclude participation in the lifestyle intervention or could significantly affect the study outcomes).

Patients with impaired fasting glucose that showed an RER indicative of glucose oxidation metabolism during fasting were matched with patients with impaired fasting glucose that showed an RER indicative of fat oxidation metabolism during fasting. Patients were matched for key variables affecting the main outcome: age, sex, baseline BMI, baseline waist circumference, baseline arm muscle area, and baseline body fat mass fraction of total body weight.

The study complied with the principles established by the Declaration of Helsinki, and written informed consent was obtained from each subject. The ethics committee of the University of Milan (n. 6/2019) approved the study procedures.

### Anthropometry

Body weight, body height, and body fat fraction of total body weight were assessed with anthropometric methods. The anthropometric measurements were collected by a well-trained registered dietitian at the ICANS center. Procedures used are detailed by Lohman and Roche [11].

Body weight was measured to the nearest 100 g with a mechanical column scale graduated to 100 g, and with a capacity of 160 kg (Seca 700, Seca GmbH, Hamburg, Germany). Body height was measured to the nearest 1 mm with a stadiometer graduated to 1 mm, and with a measuring range of 20 to 205 cm (Seca 217, Seca GmbH, Hamburg, Germany). Waist circumference was measured at the approximate midpoint between the lower margin of the last palpable rib and the top of the iliac crest, to the nearest 0.1 cm with an inextensible metric tape, 0.5 cm wide, and graduated to 1 mm (Gima 27341, Gima S.p.A., Gessate, Italy). Biceps, triceps, subscapular, and suprailiac skinfold thicknesses were measured to the nearest 0.1 mm using a skinfold caliper with a 35 mm^2^ jaw face area, exerting a 10 ± 2 g/mm^2^ pressure between the jaws, with a range of 0 to 40 mm, calibrated to 0.2 mm (Holtain Tanner/Whitehouse Skinfold Caliper, Croswell, UK).

BMI (kg/m^2^) was calculated as body weight in kilograms divided by the square of the body height in meters and classified according to the WHO guidelines [12]. Body density (kg/L) and body fat fraction of total body weight were estimated using formulas provided by Durnin and Womersley [13] and Siri [14], respectively.

### Indirect Calorimetry

Resting energy expenditure and substrate utilization were measured at baseline with indirect calorimetry.

All measurements were performed early in the morning, after a 12-hour fast, with subjects lying supine, at rest but awake, in a quiet and thermally neutral environment (24 °C). After a 15-minute resting period, O_2_ consumption and CO_2_ production were measured using the canopy dilution technique [6], with the patient wearing a transparent ventilated canopy for 30 minutes, sampling gases every 30 seconds. To avoid gas leakages, the subject's head was carefully wrapped with a veil.

Technical details of the indirect calorimeter used (Q-NRG+, Cosmed srl, Rome, Italy) are detailed by Delsoglio et al [15]. Calibration of the flowmeter and gas analyzers was performed according to the manufacturer's instructions and schedule.

For each measurement, data collected during the first 5 minutes were discarded, while data collected during the remaining minutes were averaged (mean) and scaled to provide daily resting gas exchanges. Steady state was defined as the first 5 consecutive stable 30-second readings with a coefficient of variation <10% for VO_2_ and VCO_2_, and, when available, data in steady state were preferred. The Weir formula [16] was used to estimate resting energy expenditure from gas exchanges measured at rest by indirect calorimetry.

The RER was defined as the ratio of VCO_2_ and VO_2_, and an RER < 0.775 was considered indicative of fat oxidation metabolism, while an RER > 0.925 was considered indicative of glucose oxidation metabolism. The aim was to focus on individuals with a clear predominance of either fat or carbohydrate oxidation, respectively. By selecting these more extreme values, we aimed to minimize the confounding factor of protein metabolism, which can become more significant at intermediate RER values.

### Study Size, Quantitative Variables

Sample size was calculated based on the expected difference in fasting glucose at follow-up between the fat oxidation and glucose oxidation groups (10 mg/dL). Considering a SD of fasting glucose in prediabetic patients of 15 mg/dL, we needed at least 37 patients per group with a power of 80%. We enrolled 43 patients per group.

While some quantitative data are presented also as groups defined in the literature (eg, classes of BMI, waist circumference, blood pressure, fasting glucose), we preferred to use continuous variables in the analyses. We did model the outcome as both a continuous and dichotomous variable, the latter to show the overall mean effect of the exposure and defined as reaching a fasting glucose <100 mg/dL at follow-up.

### Statistical Methods

Matching with controls was performed using coarsened exact matching, and covariate balance between groups was evaluated by calculating the standardized mean differences across imputations.

Adjusted differences between patients with glucose oxidation RER and matched patients with fat oxidation RER were computed. The differences were adjusted for all matching variables. Adjustment was performed with ordinary least squares regression or logistic regression of the outcome variable, using as independent variables the RER group and the variables to adjust for. Linearity was assumed for all adjustment variables to conserve degrees of freedom in the model, but linearity was both a reasonable assumption in relation to the outcome variable, and matching reduced model dependence. When adjusting for all matching variables, we were less concerned with conserving degrees of freedom, and more with controlling for differences due to variable binning.

To address potential confounding factors, we conducted sensitivity analyses where we additionally matched participants for menopausal status (premenopausal or postmenopausal) and for the presence or absence of metabolic syndrome. The results of these sensitivity analyses are consistent with the primary analysis and are reported in the Supplemental Appendix [17].

## Results

### Fasting RER and Glucose Metabolism

Overall, we included 10 176 patients that performed both indirect calorimetry and a complete fasting glucose profile, inclusive of insulin and glycated hemoglobin, and their characteristics are reported in Table 1. The overall sample was mainly composed of female patients (7223 (71%), and had a median (quartiles) age of 46 (36, 55) years. The median BMI was 28.3 (25.0, 32.3) kg/m^2^, but the most prevalent BMI category was that of obesity (3879, 38%), and in particular central obesity (6173 (61%)) as highlighted by the waist circumference categories. In the overall sample, 1502 (15%) and 550 (5%) of patients showed a fat oxidation and glucose oxidation metabolism at rest, respectively. The unadjusted comparison of RER groups shows differences in sex and a general worsening going from a fat oxidation to a glucose oxidation metabolism of anthropometric indices, blood pressure, and fasting glucose metabolism parameters, blood lipid profile, and liver enzymes. To account for differences in sexes [18], we also included stratified analysis in the Supplemental Appendix [17].

**Table 1.** Patients’ characteristics of the overall sample, stratified by RER categories

Characteristic	Overall N = 10 176*^a^*	Fat oxidation RER N = 1502*^a^*	Mixed RER N = 8124*^a^*	Glucose oxidation RER N = 550*^a^*	*P* value*^b^*
Sex					**<.001**
Female	7223 (71%)	1178 (78%)	5719 (70%)	326 (59%)	
Male	2953 (29%)	324 (22%)	2405 (30%)	224 (41%)	
Age (years)	46 (36, 55)	46 (35, 55)	46 (36, 55)	46 (36, 55)	.10
BMI, kg/m^2^)	28.3 (25.0, 32.3)	28.2 (24.8, 32.4)	28.3 (25.0, 32.3)	28.9 (25.6, 32.9)	**.029**
BMI categories					**.012**
Underweight	183 (1.8%)	38 (2.5%)	137 (1.7%)	8 (1.5%)	
Normal weight	2340 (23%)	363 (24%)	1874 (23%)	103 (19%)	
Overweight	3755 (37%)	540 (36%)	3011 (37%)	204 (37%)	
Obesity class I	2428 (24%)	327 (22%)	1958 (24%)	143 (26%)	
Obesity class II	996 (9.8%)	150 (10%)	777 (9.6%)	69 (13%)	
Obesity class III	455 (4.5%)	80 (5.3%)	352 (4.3%)	23 (4.2%)	
Waist circumference (cm)	96 (86, 107)	94 (84, 106)	96 (86, 107)	98 (89, 109)	**<.001**
Waist circumference categories					.10
Normal	1845 (18%)	302 (20%)	1459 (18%)	84 (15%)	
Elevated	2120 (21%)	310 (21%)	1697 (21%)	113 (21%)	
Central obesity	6173 (61%)	885 (59%)	4935 (61%)	353 (64%)	
Body fat percentage	39 (34, 43)	39 (35, 43)	39 (34, 42)	38 (33, 42)	**<.001**
Systolic blood pressure (mmHg)	120 (110, 130)	120 (110, 130)	120 (110, 130)	120 (110, 130)	**<.001**
Diastolic blood pressure (mmHg)	80 (70, 80)	80 (70, 80)	80 (70, 82)	80 (70, 85)	**<.001**
Blood pressure categories					**<.001**
Normal	5197 (57%)	841 (61%)	4102 (56%)	254 (50%)	
Elevated	386 (4.2%)	56 (4.1%)	314 (4.3%)	16 (3.1%)	
Stage 1	2823 (31%)	372 (27%)	2274 (31%)	177 (35%)	
Stage 2	779 (8.5%)	110 (8.0%)	605 (8.3%)	64 (13%)	
RER	0.83 (0.79, 0.87)	0.75 (0.73, 0.77)	0.84 (0.81, 0.87)	0.94 (0.93, 0.95)	**<.001**
Resting energy expenditure (kcal/day)	1451 (1304, 1666)	1462 (1313, 1648)	1445 (1300, 1663)	1504 (1318, 1759)	**<.001**
Glucose (mg/dL)	95 (89, 102)	94 (88, 101)	95 (89, 102)	96 (90, 104)	**<.001**
Glucose categories					**.008**
Normal	6791 (70%)	1035 (72%)	5414 (70%)	342 (65%)	
Prediabetes	2721 (28%)	368 (26%)	2188 (28%)	165 (31%)	
Diabetes	196 (2.0%)	25 (1.8%)	153 (2.0%)	18 (3.4%)	
Insulin (mIU/L)	10 (7, 15)	9 (6, 14)	10 (7, 15)	11 (8, 17)	**<.001**
HOMA-IR	2.28 (1.54, 3.59)	2.10 (1.40, 3.29)	2.30 (1.55, 3.59)	2.76 (1.80, 4.03)	**<.001**
HbA1c (%	5.44 (5.20, 5.71)	5.41 (5.17, 5.67)	5.44 (5.20, 5.71)	5.49 (5.21, 5.74)	.078
Total cholesterol (mg/dL)	205 (178, 234)	205 (176, 233)	205 (178, 234)	207 (181, 234)	.4
High-density lipoprotein cholesterol (mg/dL)	59 (49, 71)	61 (50, 73)	59 (49, 70)	56 (46, 68)	**<.001**
Low-density lipoprotein cholesterol (mg/dL)	131 (107, 158)	129 (104, 154)	131 (107, 159)	132 (110, 158)	**.017**
Triglycerides (mg/dL)	87 (63, 124)	82 (60, 117)	87 (64, 124)	97 (70, 145)	**<.001**
AST/GOT (IU/L)	18.4 (15.5, 22.4)	18.2 (15.3, 22.6)	18.4 (15.5, 22.3)	18.9 (16.0, 23.1)	.10
ALT/GPT (IU/L)	18 (14, 27)	18 (13, 26)	18 (14, 27)	20 (15, 28)	**<.001**
GGT (IU/L)	18 (12, 27)	17 (12, 26)	18 (12, 27)	18 (13, 30)	**.004**

Bold values indicate *P*-values < .05.

^a^n (%); median (Q1, Q3).

^b^Pearson's Chi-squared test; Kruskal–Wallis rank sum test.

Abbreviations: ALT, alanine aminotransferase; AST, aspartate aminotransferase; BMI, body mass index; GGT, Gamma-glutamyl transferase; GOT, Glutamate oxaloacetate transaminase; GPT, Glutamate pyruvate transaminase; HbA1c, glycated hemoglobin; HOMA-IR, Homeostatic Model Assessment of Insulin Resistance; RER, respiratory exchange ratio.

Table 2 shows the adjusted association between RER and fasting glucose metabolism. In the overall sample, a higher RER was associated with higher fasting glucose (1.7; 95% CI 0.66, 2.8; *P* = .002), higher fasting insulin, (1.9; 95% CI 1.3, 2.6; *P* ≤ .001), and consequently higher Homeostatic Model Assessment for Insulin Resistance (HOMA-IR) (0.52; 95% CI 0.34, 0.70; *P* ≤ .001).

**Table 2.** Linear regression models of glucose, insulin, and HOMA-IR

	Glucose (mg/dL)			Insulin (mIU/L)			Homeostatic model assessment		
Characteristic	Beta	95% CI	*P* value	Beta	95% CI	*P* value	Beta	95% CI	*P* value
Age (years)	0.24	0.23, 0.26	**<.001**	−0.04	−0.05, −0.03	**<.001**	0.00	−0.01, 0.00	**.005**
Sex									
Female	—	—		—	—		—	—	
Male	5.5	5.1, 6.0	**<.001**	2.3	2.1, 2.6	**<.001**	0.74	0.67, 0.82	**<.001**
BMI, kg/m^2^	0.48	0.44, 0.52	**<.001**	0.69	0.67, 0.71	**<.001**	0.18	0.18, 0.19	**<.001**
Respiratory Exchange Ratio	1.7	0.66, 2.8	**.002**	1.9	1.3, 2.6	**<.001**	0.52	0.34, 0.70	**<.001**

Bold values indicate *P*-values < .05.

Abbreviation: BMI, body mass index; HOMA-IR, Homeostatic Model Assessment of Insulin Resistance; RER, respiratory exchange ratio.

### Fasting RER and Impaired Fasting Glucose Resolution

Table 3 shows characteristics of the 86 matched impaired fasting glucose patients with a fat oxidation or glucose oxidation RER, while Table 4 shows unadjusted fasting glucose differences between baseline and follow-up in both groups. While both groups significantly reduced fasting glucose at follow-up, the fat oxidation group reduction was greater (+5.9; 95% CI 1.4, 10; *P* = .011). Consequently, the fat oxidation group showed a higher probability of normalizing fasting glycemia within 1-year of beginning the lifestyle intervention (log(odds ratio) −0.89 (95% CI −1.8, −0.03; *P* = .046)).

**Table 3.** Matched impaired fasting glucose patients with either fat oxidation or glucose oxidation RER

Characteristic	Fat oxidation RER N = 43*^a^*	Glucose oxidation RER N = 43*^a^*
Sex		
Female	29 (67%)	19 (44%)
Male	14 (33%)	24 (56%)
Age (years)	54 (44, 59)	54 (43, 64)
Body mass index (kg/m^2^)	34.1 (29.2, 37.0)	31.3 (28.6, 35.4)
Waist circumference (cm)	110 (100, 119)	104 (99, 114)
Arm muscle area (cm^2^)	56 (43, 65)	61 (47, 69)
Body fat fraction of total body weight, as %	42.7 (39.3, 45.9)	39.3 (33.7, 44.3)

^a^n (%); median (Q1, Q3).

Abbreviations: RER, respiratory exchange ratio.

**Table 4.** Unadjusted glucose differences between baseline and follow-up in the glucose oxidation and fat oxidation group

Characteristic	Baseline*^a^*	Follow-up*^a^*	Difference*^b^*	95% CI*^b^*	*P* value*^b^*
Glucose oxidation RER					
Glucose (mg/dL)	108 (103, 113)	101 (95, 111)	5.7	1.9, 9.5	**.004**
Fat oxidation RER					
Glucose (mg/dL)	107 (102, 112)	99 (91, 107)	11	7.1, 15	**<.001**

Bold values indicate *P*-values < .05.

^a^Median (Q1, Q3).

^b^Welch 2-sample t-test.

Abbreviation: RER, respiratory exchange ratio

Finally, Table 5 show intragroup differences in anthropometric parameters between baseline and follow-up: both groups were able to reduce body weight, BMI, waist circumference, and body fat fraction of total body weight. Although comparing anthropometric parameters at follow-up between the RER groups (Table 6) shows that while differences in weight loss were similar in the 2 groups (−0.39; 95% CI −3.5, 2.7; *P* = .8), body fat was higher at follow-up in the glucose oxidation group (+1.2; 95% CI 0.07, 2.4; *P* = .038).

**Table 5.** Intragroup differences in anthropometric parameters between baseline and follow-up

Characteristic	Baseline*^a^*	Follow-up*^a^*	Difference*^b^*	95% CI*^b^*	*P*-value*^b^*
Body weight (kg)					
Fat oxidation RER	93 (80, 111)	85 (70, 104)	5.3	2.7, 8.0	**<.001**
Glucose oxidation RER	88 (81, 106)	82 (76, 99)	5.7	3.9, 7.5	**<.001**
Body mass index (kg/m^2^)					
Fat oxidation RER	34.1 (29.2, 37.0)	30.8 (26.5, 34.1)	2.0	1.1, 2.9	**<.001**
Glucose oxidation RER	31.3 (28.6, 35.4)	29.4 (26.4, 32.7)	2.1	1.4, 2.8	**<.001**
Waist circumference (cm)					
Fat oxidation RER	110 (100, 119)	99 (90, 111)	6.2	4.6, 7.8	**<.001**
Glucose oxidation RER	104 (99, 114)	100 (94, 108)	5.4	3.7, 7.1	**<.001**
Body fat fraction of total body weight, as %					
Fat oxidation RER	43 (39, 46)	40 (37, 44)	2.6	1.7, 3.4	**<.001**
Glucose oxidation RER	39 (34, 44)	39 (30, 42)	1.6	0.79, 2.4	**<.001**

Bold values indicate *P*-values < .05.

^a^Median (Q1, Q3).

^b^Paired t-test.

Abbreviation: RER, respiratory exchange ratio.

**Table 6.** Baseline adjusted differences in anthropometric parameters at follow-up between the glucose oxidation and fat oxidation group

	Body weight (kg)			Body mass index (kg/m^2^)			Waist circumference (cm)			Body fat fraction of total body weight, as %		
Characteristic	Beta	95% CI	*P* value	Beta	95% CI	*P* value	Beta	95% CI	*P* value	Beta	95% CI	*P* value
Baseline value	0.98	0.89, 1.1	**<.001**	1.0	0.90, 1.1	**<.001**	0.93	0.85, 1.0	**<.001**	1.1	1.0, 1.2	**<.001**
RER categories												
Fat oxidation RER	—	—		—	—		—	—		—	—	
Glucose oxidation RER	−0.39	−3.5, 2.7	0.8	−0.09	−1.2, 1.0	.9	0.72	−1.5, 2.9	.5	1.2	0.07, 2.4	**.038**

Bold values indicate *P*-values < .05.

Abbreviation: RER, respiratory exchange ratio.

The sensitivity analyses, which included additional matching for menopausal status and metabolic syndrome status, yielded results similar to those of the primary analysis. These findings are detailed in the Supplemental Appendix [17].

## Discussion

In this study, we demonstrate that extreme ranges of fasting RER (RER < 0.775 and RER > 0.925) are indicative of glucose metabolism and metabolic flexibility. Furthermore, we found that patients with impaired fasting glucose exhibiting a fat oxidation RER are more likely to normalize fasting glycemia within 1 year of being prescribed a lifestyle intervention than those with a glucose oxidation RER. While the fat oxidation group experienced greater fasting glucose reduction, weight loss was similar between the 2 groups, albeit with a greater fraction of lean mass loss in the glucose oxidation group.

The relationship between metabolic flexibility and glucose metabolism has already been established [19-21]. To our knowledge, the use of fasting RER to describe this relationship at an individual level has not yet been pursued. Fasting RER, an easily obtainable parameter routinely collected in nutritional clinical centers, has thus the potential to become a widely used tool for detecting a patient's metabolic flexibility status. Nonetheless, fasting RER has several shortcomings when compared with reference methods. Firstly, it can change in an individual due to factors other than metabolic flexibility. The duration of the fast is a major determinant, as prolonged fasting promotes a shift towards fat oxidation and a consequent decrease in RER [22]. Additionally, dietary habits, particularly a high-carbohydrate diet, can influence RER by initially sustaining glycogen stores and carbohydrate utilization [23]. Our findings from a large cohort of over 10 000 individuals show a clear association between higher fasting RER and glucose metabolism alterations commonly observed in metabolically inflexible patients. As detailed in Table 2, a higher RER was associated with higher fasting glucose, higher fasting insulin, and consequently higher HOMA-IR. These associations suggest that even a single fasting RER measurement can provide valuable insights into an individual's metabolic health and risk for prediabetes. This is consistent with other studies that have found a prognostic value of high fasting RER in relation to fat gain and metabolic health [24, 25].

Current research indicates a strong association between impaired metabolic flexibility and prediabetes. Prediabetic individuals often exhibit a reduced ability to switch between glucose and fatty acid oxidation, contributing to elevated blood glucose levels and insulin resistance [2]. This metabolic inflexibility arises from a complex interplay of factors, including insulin resistance, mitochondrial dysfunction, and lifestyle choices [26]. The inability to efficiently utilize both glucose and fatty acids not only increases the risk of progression to type 2 diabetes but also elevates the risk of other comorbidities [27]. Our study confirms that metabolic flexibility, as indicated by fasting RER, is associated with both impaired fasting glucose resolution and improvements in fasting glucose. We found that individuals with a lower fasting RER, suggesting a greater ability to utilize fats for energy, were more likely to achieve normal fasting glucose levels after a lifestyle intervention.

In this paper, we show that metabolic inflexibility can significantly impact the quality of weight loss, potentially leading to a greater loss of fat-free mass during a diet. Studies have shown that individuals with metabolic inflexibility exhibit reduced fat oxidation rates, even during fasting conditions or low-carbohydrate diets, leading to increased fat accumulation and difficulty losing weight [28]. This preference for glucose as the primary fuel source can also result in muscle protein breakdown, further compromising body composition and metabolic rate [29].

This study provides valuable insights into the relationship between metabolic flexibility and diabetes resolution. A key strength lies in the large sample size, which allowed for precise matching of impaired fasting glucose patients based on their metabolic profiles and enabled the investigation of clinically relevant outcomes like diabetes remission. Furthermore, the detailed characterization of participants' nutritional status at baseline strengthens the study's ability to identify factors associated with metabolic health. However, the lack of a reference method to directly assess metabolic flexibility, such as an insulin clamp or incremental exercise testing with indirect calorimetry, represents a limitation. Relying solely on fasting measures may not fully capture the dynamic changes in substrate utilization that characterize metabolic flexibility. Nonetheless, this pragmatic approach enabled the inclusion of a large cohort, providing valuable real-world data on the association between fasting metabolic profiles and long-term health outcomes. Moreover, we selected patients solely based on fasting glucose, and the assessment of glycated hemoglobin could have provided a greater understanding of the prediabetes phenotype [30]. The overnight fast was used to standardize the measurement condition, although diet prior the assessment was not standardized. RER and insulin were not systematically re-evaluated at follow-up, limiting our ability to assess changes in metabolic flexibility and glycemic control over time. Additionally, fatty acid levels were not analyzed in this study, precluding a more comprehensive assessment of metabolic profiles. Fat was estimated using anthropometric measurements rather than reference methods.

In conclusion, this study shows that fasting RER, a readily accessible clinical measure, is a valuable indicator of glucose metabolism and can predict the likelihood of impaired fasting glucose resolution with lifestyle intervention. While fasting RER may be associated with metabolic flexibility, further research is needed to directly assess this relationship. Nonetheless, our findings highlight the potential of fasting RER as a practical and scalable tool for assessing metabolic health and guiding personalized lifestyle interventions in large populations.

## Disclosures

The authors of this article declare that they have no conflicts of interest.

## Data Availability

Restrictions apply to the availability of some or all data generated or analyzed during this study to preserve patient confidentiality or because they were used under license. The corresponding author will on request detail the restrictions and any conditions under which access to some data may be provided.

## Abbreviations

BMI: body mass index
HOMA-IR: Homeostatic Model Assessment for Insulin Resistance
RER: respiratory exchange ratio
VCO_2_: carbon dioxide produced
VO_2_: oxygen consumed
